# Malaria elimination strategy and challenges in People’s Republic of China

**DOI:** 10.1186/1475-2875-11-S1-P138

**Published:** 2012-11-09

**Authors:** Xiao-Nong Zhou, Shu-Shen Zhou, Xia Zi-Guang

**Affiliations:** 1 National Institute of Parasitic Diseases, China CDC, Shanghai 200025, P.R. China

## Background

Malaria remains an infectious disease of foremost public health importance in the People’s Republic of China. Historically, high malaria incidence rates have been reported from 24 provinces of P.R. China with more than 30 million cases annually reported. With the significant reduction of malaria incidence, the national malaria elimination was launched in 2010.

## Methods

The risk factors related to malaria transmission in China was reviewed based on the previous literature reviewing, and the capacity of malaria elimination was analysed based on the readiness in surveillance and response system. The challenges and future research priorities related to the elimination strategy were put forward.

## Results

Owing to large-scale control activities facilitated through primary healthcare networks and community participation, the infection rate of *Plasmodium vivax* has been reduced to under 0.01% in most areas of China, and *P. falciparum* malaria has been eliminated in most provinces, except Yunnan and Hainan. The elimination strategy formulation and its readiness analysis were performed with discussion on the challenges for the national malaria elimination programme in China, while the sourthern border areas in Yunnan will be the one of most hard issue to elimination the disease. Finally the recommendation on surveillance and response approaches based on currently satus leading to malaria elimination in China were put forward.

## Conclusion

The national malaria elimination programme launched in 2010 is able to achieve its optimal goal to eliminate malaria in whole country by 2020, if facilitated by surveillance and response system.

